# Scenario-based occupational risk assessment for power grid maintenance workers

**DOI:** 10.1371/journal.pone.0354455

**Published:** 2026-08-05

**Authors:** Wan Wang, Xinghao Zhao, Lei Wang, Tingxin Qin

**Affiliations:** 1 Department of Public Safety, China National Institute of Standardization, Beijing, P. R. China; 2 Training Department No. 2, Cadre Training College of the Ministry of Emergency Management of the People’s Republic of China, Beijing, P. R. China; G H Raisoni College of Engineering and Management, Pune, INDIA

## Abstract

**Background:**

Safety is the fundamental guarantee of power production. The safety of power operation personnel is closely related to public welfare and the sustained and stable development of the power industry. However, factors endangering the safety of power operation personnel cover multiple dimensions, including personnel operation, equipment status, working environment, and management system, which pose considerable challenges to the comprehensiveness and accuracy of risk identification.

**Methods:**

This study integrates scenario construction theory into power operation practice. Based on the grey relational degree and Bayesian network method, a risk assessment method suitable for power operation personnel is constructed and applied in typical power operation scenarios.

**Results:**

In this paper, risk assessment methods are used to identify risk factors, calculate their weights, and conduct logical analysis for the operation of live-line connecting branch leads on 10 kV overhead lines. Through scenario construction, the operation is divided into four key stages with distinct dominant risk factors in each stage. The results show that human factors and environmental factors account for relatively high overall weights, which is consistent with the pattern of actual accidents. Among them, the impact of operators’ physical and mental health is unexpected and requires special attention. Meanwhile, there is a significant risk transmission effect among procedures, and the quality of preceding procedures directly determines the safety of subsequent operations.

**Conclusions:**

The calculated results provide direct data support and a reference for decision-making on the precise control of power operation safety, thereby improving the scientific rigor and effectiveness of safety prevention and control measures for power operations.

## 1. Introduction

Electrical work is a crucial foundation for ensuring the stable operation of the power system, directly impacting the safety of workers and national property. Currently, the factors threatening the safety of electrical workers are complex, encompassing multiple dimensions such as personnel operation, equipment status, site environment, and management mechanisms, posing significant challenges to the accuracy of risk identification and the effectiveness of prevention and control measures. Accurately identifying and deeply analyzing various risk factors affecting electrical work safety would provide more comprehensive protection for workers’ personal safety. However, existing studies mainly focus on the macro-level safety management and inherent risks of power systems. [1–11] Sadeghi-Yarandi et al. developed a novel Electrical Industry Safety Risk Index for the electricity power distribution industry based on fuzzy analytic hierarchy process and conducted comparative research [1]; Lee et al. proposed a safety autonomous platform for data-driven risk management based on an on-site AI engine in the electric power industry, and implemented the platform architecture and performed performance verification [2]; Efthymios Karangelos and Louis Wehenkel put forward an integrated cyber-physical risk management framework for electric power transmission grid security and carried out relevant optimization analysis [3]; Acakpovi and Dzamikumah adopted questionnaires and in-depth interviews to investigate the compliance of occupational health and safety management systems in a hydroelectric power plant in Ghana and sorted out safety influencing factors [4]; Shao Guangzheng constructed a scenario analysis model for large-scale blackout events, analyzed the evolution process of events from multiple dimensions and verified the model with field drills [5]; Sroka and Złotecka assessed the risk of large blackout failures and vulnerability of power systems using the bow tie model based on historical statistical data, and analyzed the impact of power reserve deficit [6]; Alhelou et al. presented a comprehensive survey on power system blackouts and cascading events over the past decade, summarized accident causes, analysis methods and existing problems, and proposed future research directions [7]. Wang et al. conducted a series of studies on risk and reliability assessment of overhead contact lines (OCLs). They first developed a data-driven lightning-related failure risk prediction method by integrating a Bayesian network with a spatiotemporal fragility model to characterize the relationship between lightning strikes and OCL failures and support predictive maintenance decisions [8]. Building upon this work, a dynamic Bayesian network-based predictive probabilistic risk analytics framework was proposed to identify critical risk factors and model the dynamic propagation of weather-driven risks, considering system failures, economic losses, and social impacts simultaneously [9]. To further enhance reliability assessment, the authors established a data-driven time-dependent reliability prediction framework that incorporates lightning strikes, imperfect maintenance, and common-cause failures, enabling dynamic reliability evaluation under evolving operational conditions [10]. More recently, an uncertainty-aware trustworthy weather-driven failure risk predictor based on probabilistic deep multitask learning and deep Gaussian processes was developed to simultaneously predict multiple weather-induced failures while quantifying epistemic and aleatory uncertainties, thereby improving the reliability and interpretability of risk prediction results [11]. From the above review, detailed research targeting workers’ personal safety and potential safety hazards in actual power operation scenarios remains insufficient. Few studies have conducted in-depth exploration of on-site safety issues faced by power operation personnel.

Therefore, given that targeted studies on power workers’ safety and operational risk assessment are still inadequate, this paper centers on power personnel safety and develops a dedicated risk assessment method for power operation scenarios. This method innovatively combines scenario construction theories, Bayesian network (BN), Job Safety Analysis (JSA) and Grey Correlation Analysis (GCA) [8,12–15]. It integrates qualitative analysis with quantitative calculation, as well as subjective judgment with objective evaluation, which makes it different from conventional assessment methods. On this basis, we establish typical live-line work scenarios and conduct quantitative calculations on risk factors at key links, so as to fully validate the feasibility of the newly developed method.

## 2. Materials and methods

### 2.1 Overview of risk assessment methods

**Fig 1:**
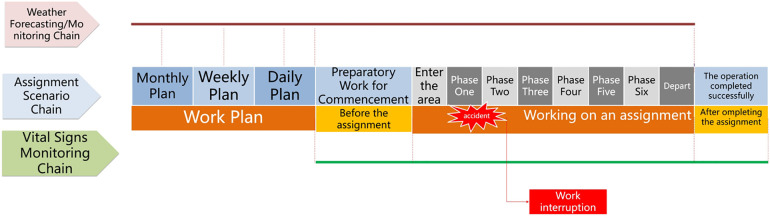
Schematic diagram of power operation implementation scenario.

Fig 1 illustrates the detailed procedures of power operation work. Power work advances through a sequence of stages: monthly, weekly, and daily planning phases; pre-operation preparation and site entry; the operational phase, consisting of various sub-phases; and ultimately, completion and exit from the site, signifying the successful conclusion of the operation. Due to the potential for unforeseen events arising from factors such as personnel, materials, environment, and management, it is essential to conduct concurrent weather forecasting and monitoring, along with vital sign assessment, throughout the entire operational process to dynamically evaluate environmental and personnel conditions. Nonetheless, unforeseen interruptions to the operation may arise at any phase.

**Fig 2:**
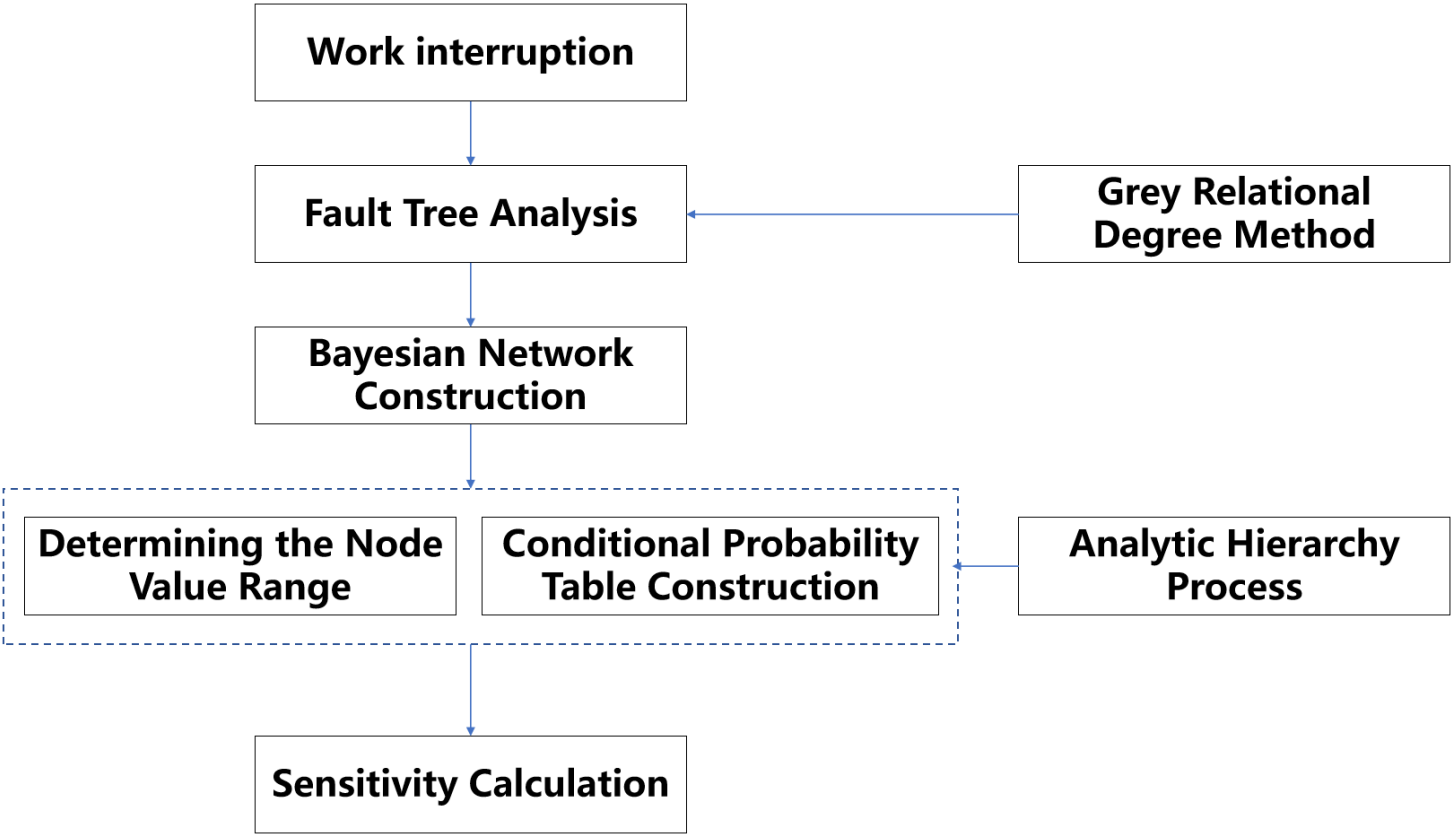
Risk assessment method for power workers.

To accurately identify and assess the degree of risk impact, a risk assessment method for power workers was developed (see Fig 2). The specific process is as follows: After identifying the research object, relevant cases and data are collected simultaneously; referring to previous research results and comparing various risk assessment methods, [16–23] the main risk factors affecting the occurrence of accidents are identified using analysis methods such as grey relational analysis, and then a fault tree (FT) is reasonably constructed. Subsequently, based on expert experience, the impact weights of risk factors are scored, and the value range and conditional probability table of each node in the FT are clarified. On this basis, the BN method is used to perform calculations to obtain the sensitivity of different risk factors at each stage, and then the ranking of the degree of impact of risk factors is obtained.

### 2.2 Grey relational degree calculation method

Grey system theory takes “small sample” and “poor information” uncertain systems as its research objects. It mainly extracts valuable information by generating and developing known information. Grey relational analysis refers to analyzing the relationship between multiple factors in a grey system. It is an important part of grey system theory and methods. Current research mainly uses three perspectives to define grey relational degree: “distance, slope, and slope difference,” which correspond to Deng’s relational degree, absolute relational degree, and T-type relational degree, respectively. This paper adopts the most classic Deng’s relational degree calculation method, which reflects the degree of relation between two sequence curves by calculating the average distance between them. The specific calculation process is as follows. [24]

1) Determine the reference sequence and comparison sequence:

$$\begin{array}{c} X_{\text{0}}\left(x_{\text{0}}(\text{1}),x_{\text{0}}(\text{2}),\ldots ,x_{\text{0}}(n)\right)\;(i=\text{0},\text{1},\ldots m) \end{array}$$ (1)

$$\begin{array}{c} X_{i}\left(x_{i}(\text{1}),x_{i}(\text{2}),\ldots ,x_{i}(n)\right)\;(i=\text{0},\text{1},\ldots m) \end{array}$$ (2)

Where X_0_ represents the reference sequence, which in this paper refers to the sequence of the total number of accidents; X_i_ represents the comparison sequence, which in this paper refers to the sequence of the number of accidents caused by the i-th risk factor; m represents the type of risk factor; and n represents the time.

2) Determine the difference sequence of the given sequence, i.e.,

$$\begin{array}{c} \Delta_{i}(k)=\left|x_{\text{0}}(k)-x_{i}(k)\right| \end{array}$$ (3)

$$\begin{array}{c} \Delta_{i}=\left(\Delta_{i}(\text{1}),\Delta_{i}(\text{2}),\ldots \Delta_{i}(n)\right),\;(i=\text{0},\text{1},\ldots m) \end{array}$$ (4)

3) Determine the maximum and minimum differences between the two poles, i.e.,

$$\begin{array}{c} \Delta_{max}={max}_{i}{max}_{i}\Delta_{i}(k) \end{array}$$ (5)

$$\begin{array}{c} \Delta_{min}={min}_{i}{min}_{i}\Delta_{i}(k) \end{array}$$ (6)

4) Determine the correlation coefficient of the data column, i.e.,

$$\begin{array}{c} \delta_{i}(k)=\frac{\Delta_{min}+\rho \Delta_{max}}{\Delta_{i}(k)+\rho \Delta_{max}}\;\rho \varepsilon (\text{0},\text{1}) \end{array}$$

$$\begin{array}{c} k=\text{1},\text{2},\ldots ,n;i=\text{1},\text{2},\ldots ,m \end{array}$$ (7)

Where ρ is the resolution coefficient, and its value ranges from (0, 1), and it is generally taken as ρ = 0.5.

### 2.3 Analytic hierarchy process (AHP) for calculating prior probabilities

The expert scoring method was used as the prior probability for Bayesian calculation. An expert scoring questionnaire was designed, which mainly included the basic information of the experts, the probability of occurrence of risk factors (see Table 1), and the probability of occurrence of events (see Table 2). Considering the experts’ education, professional title, and work experience, the AHP was used to measure the weight of each piece of information. The comprehensive weight of each expert was obtained by simple summation and normalization (see Table 3),The comprehensive weight of each expert was obtained by simple summation and normalization (see Table 3). The consistency ratio CR of this expert judgment matrix is approximately 0.0032, which is far less than 0.1, indicating that the judgment matrix has excellent consistency and the weights are valid. The probability of occurrence of each risk factor and the probability of occurrence of intermediate events were calculated, as shown in formula (6).

**Table 1:** Probability of risk factor occurrence.

Risk level	Description of the probability of occurrence	Probability of occurrence
1	Almost impossible	0.0002
2	Less frequently	0.002
3	Occasionally	0.02
4	Frequently	0.2
5	Massive occurrence	0.9

**Table 2:** Probability of event occurrence (example).

Long service life of charged parts	Bad weather	Possibility of leakage from a charged body (0–10)
T	T	
T	F	
F	T	
F	F	

Note: T represents occurrence, and F represents non-occurrence.

**Table 3:** Expert information weighting.

Expert Information Classification	Weight	Information Segmentation	Weight
Work experience	0.58	5 years (inclusive) or less	0.04
		5-15 years (inclusive)	0.13
		15 years or more	0.41
Education	0.11	High school (or vocational school)	0.01
		Bachelor’s degree (Associate’s degree)	0.03
		postgraduate	0.07
Technical title	0.31	primary	0.02
		intermediate	0.05
		Technician and above	0.24
CR ≈ 0.0032			

For valid questionnaires, calculate the probability of each risk factor occurring in each stage:


$$\begin{array}{c} P=\sum_{i=\text{1}}^{n}W_{i}P_{i}\times \text{1}\text{0}\text{0}\% \end{array}$$ (8)

Where W_*i*_ is the overall weight of each expert, P_*i*_ is the probability of the risk factor occurring as perceived by the experts, and n is the number of experts.

### 2.4 Bayesian network (BN) computation method

BN is an effective method for probabilistic reasoning and decision analysis. According to Bayes’ theorem, if A and B are two random events, given that A has already occurred, the posterior probability of event B occurring can be determined as follows:


$$\begin{array}{c} P\left(B_{i}|A\right)=\frac{P\left(B_{i}\right)P\left(A|B_{i}\right)}{\sum_{i=\text{1}}^{S}P\left(B_{i}\right)P\left(A|B_{i}\right)} \end{array}$$ (9)

Where P$\left(B_{i}|A\right)$ is the probability of event B_*i*_ occurring given that event A has occurred;

P$\left(A|B_{i}\right)$ is the probability of event A occurring given that event B_*i*_ has occurred;

P(B_*i*_) is the prior probability of event B_*i*_ occurring.

Using Netica 32 software, the posterior probability and sensitivity of the root node are calculated according to formula (10). Sensitivity index, representing the relative change rate of the posterior probability compared with the prior probability after introducing risk factors, thus highlighting the magnitude of the impact exerted by the specific risk factor.


$$\begin{array}{c} S=\frac{P(posterior)-P(prior)}{P(prior)} \end{array}$$ (10)

Where S is the sensitivity index;

P(posterior) is the posterior probability;

P(prior) is the prior probability.

This method is applicable to all types of power operations. Firstly, case analysis and grey relational analysis are used for objective calculation to identify the core risk factors of operations. Secondly, the entire workflow is sorted out and refined based on scenario construction theory. Thirdly, fault trees are established for each operation stage, and combined qualitative and quantitative risk analysis is performed on each work unit. Finally, a logic library of cause-effect relationships is established.

### 2.5 Ethics statement

This research collected scoring opinions from professional experts only and does not involve human clinical participants, medical records or human samples. All experts were fully informed of the study content and confidentiality rules before participation, and all scoring data was fully anonymized without retaining identifiable personal information. No minors took part, and no clinical informed consent procedures were applicable here.

In addition to expert scoring data, this study also adopts case materials collected from publicly accessible official open information websites. All retrieved case data are publicly released resources without access control requirements, and the whole process of data collection, sorting and statistical analysis strictly complies with the terms of service, data usage specifications and relevant copyright rules of each source website. No confidential, internal or restricted information was captured; all case records contain no sensitive personal identifiable information. The data acquisition and analytical workflow fully meet the official data access requirements of all platforms we sourced cases from.

## 3. Results

### 3.1 Analysis of risk factors for live-line work

Using live-line work as an example, scenario creation was conducted. First, we collected historical cases of electric shock accidents over the past 30 years from public websites and finally compiled more than 1,000 case records, including both the direct and indirect causes of each accident. Risk factors were analyzed in four dimensions: personnel, equipment, environment, and management. According to the case statistics, the data are shown in Table 4. The gray relational degree calculation method was employed to determine the correlation degree ranking of hazards in each dimension (refer to Table 4). Additional subdivision and computation of correlation degrees were conducted for each facet. The resolution coefficient ρ = 0.5 was used, and the correlation degree of the risk factors influencing electric shock incidents in power operations was computed using formula (5). Thirteen risk factors have a high degree of connection. The precise outcomes are presented in Table 5.

**Table 4:** Factors influencing safety incidents.

Risk Factors	Tier 1 risk factors	Secondary risk factors	Level 3 risk factors	Frequency	Total
Human factors	vital signs	Health condition abnormal	/	4	824
		Psychological abnormality	Risk-taking mentality	6	
			Other psychological abnormalities	4	
	Behavioral risks and harmful factors	Command error	Command error	13	
			Violation command	29	
		Operational error	Misoperation	72	
			Illegal operation	398	
			Other operational errors	1	
		Protection issues	Unprotected	47	
			Improper protection	20	
			Insufficient protection distance	9	
		Monitoring error	/	210	
		Other behavioral risks and Harmful factors	/	11	
Equipment factors	Physical hazards and harmful factors	Defects in equipment, facilities, tools and accessories	Insufficient strength	11	218
			Insufficient stiffness	1	
			Poor stability	7	
			Poor seal	1	
			Poor corrosion resistance	2	
			brake defect	1	
			Equipment, facilities and tools attached Other defects	18	
		Protective defects	Defects in protective devices and facilities	53	
			Improper protection	68	
			Other protective defects		
		Electrical injury	Charged part exposed	28	
			leakage	27	
			Other electrical injuries	1	
Environmental factors	Indoor Workplace Poor environment	/	Clutter indoor workplace	9	31
			Narrow indoor workplace	1	
			Slippery indoor floor	1	
			Unsuitable indoor temperature, humidity and air pressure	3	
			Other poor environmental conditions of indoor work areas	3	
	Outdoor Workplace Poor environment	/	Severe weather and environmental conditions	12	
			Narrow/cluttered work site	1	
			Other adverse environmental conditions of outdoor work areas	1	
			Incomplete organizational structure	29	
			Unimplemented accountability system	141	418
Management factors	/	/	Inadequate rules and regulations	248	

**Table 5:** Correlation of risk factors for electric shock accidents.

Risk factors	Correlation	Relevance Ranking
Human factors	0.958	1
Equipment factors	0.879	2
Environment factors	0.863	4
Management factors	0.865	3

**Fig 3:**
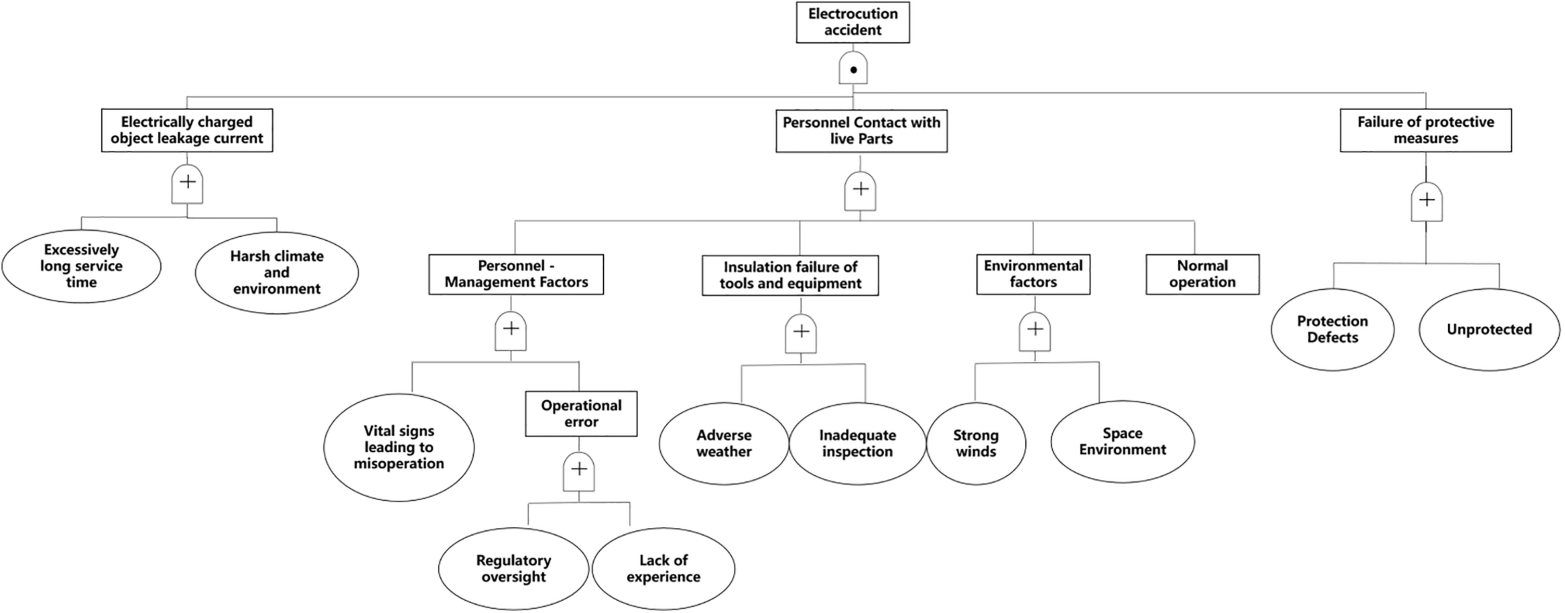
Fault tree of electric shock accidents during power operations.

Based on the calculation results in Table 6, an FT was constructed for electric shock accidents during power operations, as shown in Fig 3. It should be noted that there were considerable controversies over management factors during the investigation, so they were temporarily excluded from the fault tree analysis. Electric shock accidents only occur when leakage from a live conductor, contact with a live conductor, and failure of protective measures all occur simultaneously. An electric shock may be caused by one or more risk factors, including human error, environmental factors, tool malfunction, or other causes. Leakage from a live conductor may be due to prolonged service time or long-term harsh environments and climates. Contact with a live conductor sometimes occurs during normal work, mostly due to improper operation or tool malfunctions. Analyzing human, material, environmental, and management factors, human factors mainly include problems with the vital signs of power workers or violations/misoperations; environmental factors mainly include harsh environments and climates, as well as cluttered or confined spaces; failure of protective measures may be due to inadequate protection, including wearing unsuitable protective clothing or safety belts, problems with the protective clothing or safety belts themselves, or even the absence of task protection.

**Table 6:** Ranking of the correlation between major sub-risk factors.

Risk Factors	Secondary risk factors (Level 3 risk factors for environment and management factors)	Correlation
Human factors	Health condition abnormal	0.002
	Psychological abnormality	0.004
	Command error	0.032
	Operational error	0.643
	Protection issues	0.104
	Monitoring error	0.157
	Other behavioral risks and Harmful factors	0.016
Equipment factors	Defects in equipment, facilities, tools and accessories	0.307
	Protective defects	0.348
	Electrical injury	0.224
Environment factors	Clutter indoor workplace	0.157
	Narrow indoor workplace	0.157
	Severe weather and environmental conditions	0.471
	Other adverse environmental conditions of outdoor work areas	0.078
Management factors	Incomplete organizational structure	0.062
	Unimplemented accountability system	0.031
	Inadequate rules and regulations	0.772

### 3.2 Construction of typical live-line work electric shock accident scenarios

Using “live-line connection of a 10 kV overhead line” as a typical live-line work scenario, the key stages of this operation include the voltage testing stage, the branch line confirmation stage, the insulation shielding stage, the branch line connection stage, and the insulation shielding removal stage. Insulation shielding involves the electrician positioning the insulating bucket appropriately and applying insulation shielding measures to the near-side phases, following the sequence from near to far, from bottom to top, and from live to grounded conductors. Since the risk factors for the insulation shielding setup and removal stages are almost identical, these two stages can be combined, and the entire process is analyzed and the scenario constructed according to four stages.

**Fig 4:**
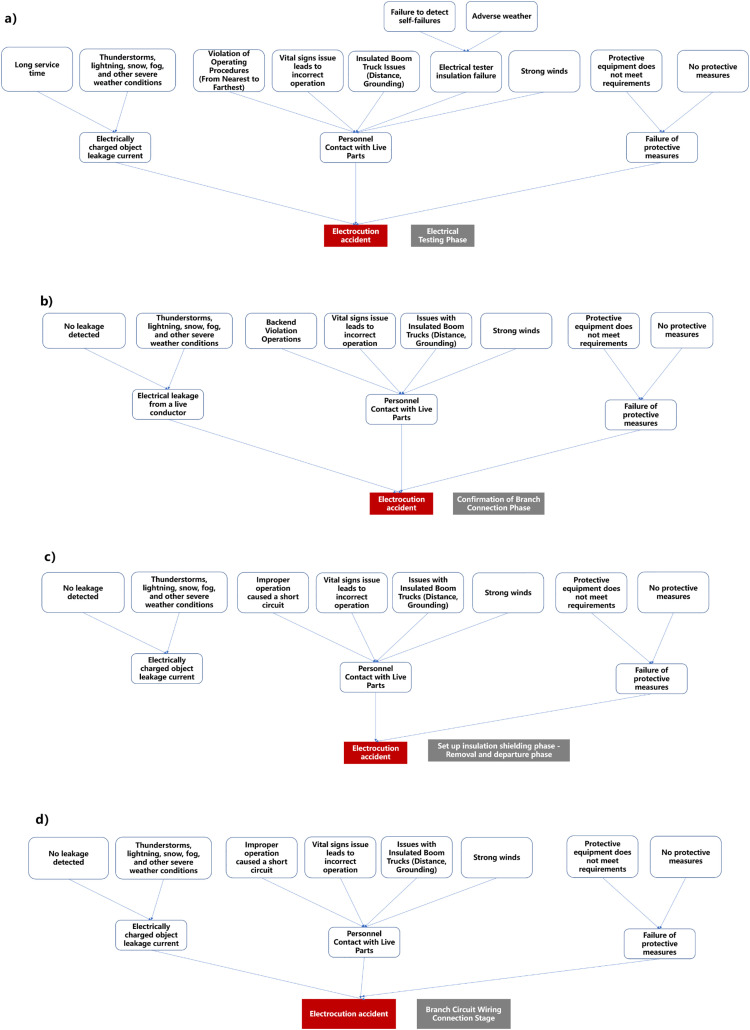
Schematic diagram of risk factors affecting each stage of “Live connection of 10 kV overhead line branch line lead”: a) Voltage testing stage; b) Branch line confirmation stage; c)Setting up insulation shielding and dismantling and leaving stage; d) Branch line splicing stage.

Based on the FT model shown in Fig 3, combined with case analysis and field survey results, a specific evolution path analysis of risk factors in each operation stage was conducted. The detailed process and conclusions are shown in Fig 4. A systematic review revealed that the core differences and key hidden dangers of risks in each stage are mainly reflected in the following aspects: In the voltage testing stage, the insulation failure of the voltage detector, a core safety verification step before electrical work, is one of the important risks at this stage. If the voltage detector fails to meet insulation performance standards due to insulation aging, damage, or mismatch between the selected type and the actual voltage level, it will not only directly affect the accuracy of the voltage testing results but may also cause misjudgment of liveness, laying the foundation for safety hazards in all subsequent operations. Simultaneously, if leakage problems in live parts are not effectively detected during the voltage testing stage, this hidden risk will further extend to the stage of confirming branch lines. Combined with potential violations by downstream personnel (such as failing to verify line numbers according to procedures or conducting verification work without reconfirming the line’s de-energized status), these factors will significantly increase the probability of electric shock accidents at this stage. During the installation or removal of insulation shielding, the risks primarily lie in improper operation at each stage. For example, insulation shielding equipment may not be used according to specifications and voltage levels; the shielding area may not completely cover all exposed live parts; and the removal of shielding may not follow established procedures, such as removing shielding from near to far and from bottom to top. These oversights can directly expose live areas, exposing workers to the risk of accidental contact. In the final branch connection stage, the hidden dangers left over from inadequate insulation shielding in the previous stage are the most prominent issues causing electric shock accidents at this stage and require close attention. Besides the core differences, weather factors, operational errors, and protective malfunctions are common risk points at all stages.

### 3.3 Calculation of the degree of influence of risk factors

**Fig 5:**
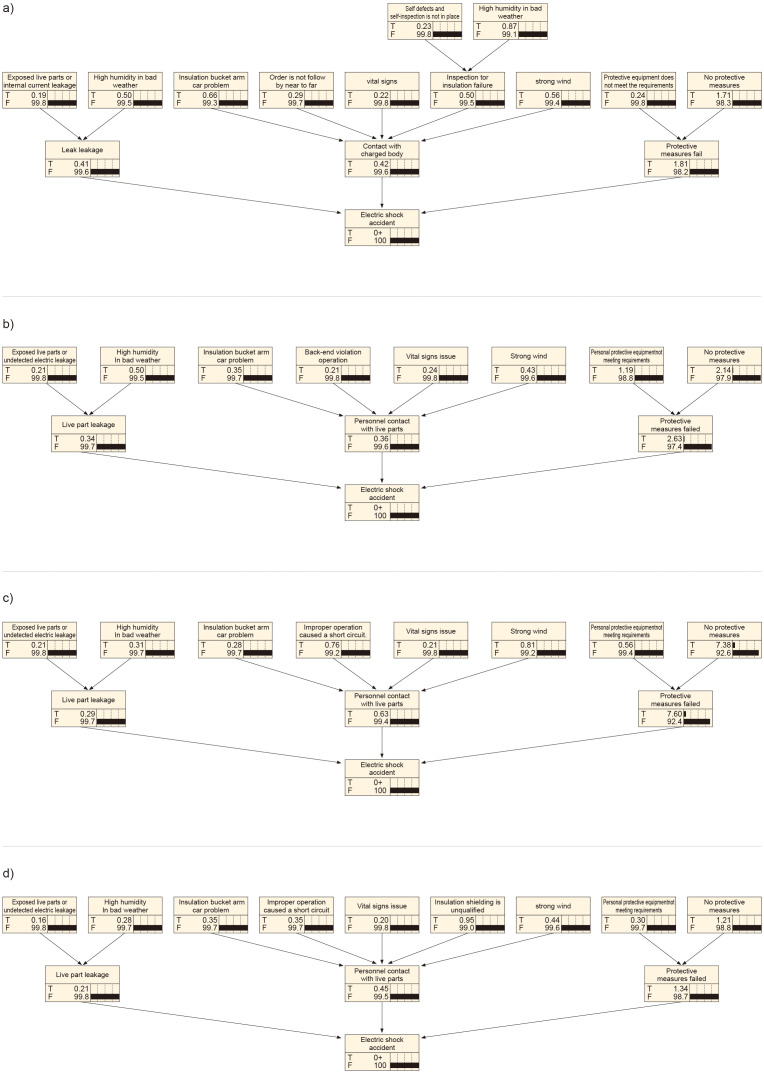
Bayesian network prior probability distribution diagram: a) Voltage testing stage; b) Branch line confirmation stage; c)Setting up insulation shielding and dismantling and leaving stage; d) Branch line splicing stage.

**Fig 6:**
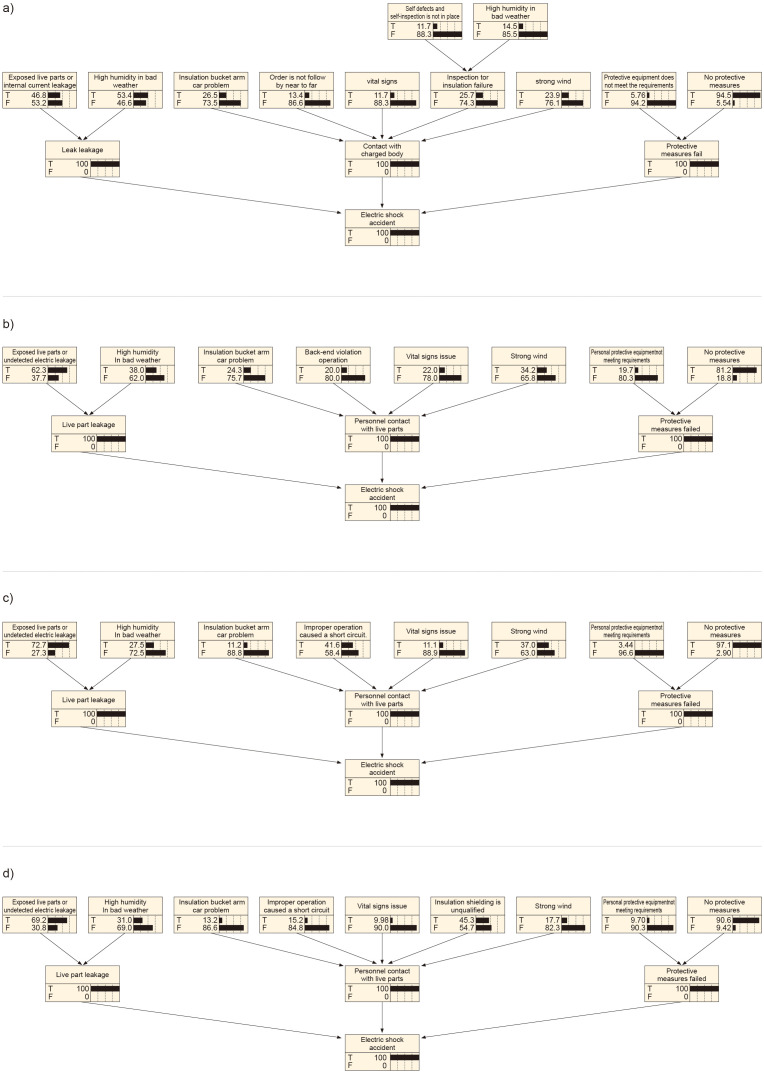
Bayesian network posterior probability diagnosis diagram: a) Voltage testing stage; b) Branch line confirmation stage; c)Setting up insulation shielding and dismantling and leaving stage; d) Branch line splicing stage.

Fig 4 was transformed into a BN (Figs 5 and 6), and 33 experts were invited to score it. A total of 37 valid questionnaires were collected. Based on the expert scores, the magnitude of the impact of risk factors and the probability of events occurring at each stage were calculated. The magnitude of the impact of risk factors at each stage is shown in Table 7. The calculation results show that the degree of impact of different risk factors varies at each stage. The risk factor with the greatest impact at the voltage detection stage is leakage or exposure to live parts. The risk factor with the greatest impact at the other three stages is the failure to detect leakage. Moreover, the sensitivity ranking of each risk factor also varies at each stage.

**Table 7:** Impact of risk factors at rach stage.

Number	Basic events	Prior probability (%)	Posterior probability (%)	Sensitivity	Sensitivity Ranking
Electrical testing stage					
1	A charged component has been in service for a long time, causing leakage	0.1926	0.468	242	1
2	Leakage caused by severe weather conditions, such as thunderstorms, lightning, snow, fog, and rain	0.4984	0.534	106	2
3	The order of work was not from nearest to furthest	0.2856	0.134	46	6
4	vital signs issues	0.2162	0.117	53	4
5	insulated bucket truck issues (distance, grounding)	0.6629	0.265	39	8
6	Inherent defects and inadequate self-testing led to insulation failure of the testing equipment	0.2347	0.117	49	5
7	Severe weather caused insulation failure of the detector	0.8663	0.145	16	10
8	has strong winds	0.5612	0.239	42	7
9	Protective equipment does not meet requirements	0.2391	0.0576	23	9
10	has no protective measures	1.7108	0.945	54	3
Confirming branch line stage					
1	leakage current from the charged body was not detected	0.2074	0.623	299.39	1
2	Leakage caused by severe weather conditions, such as thunderstorms, lightning, snow, fog, and rain	0.4980	0.380	75.305	5
3	back-end illegal operation	0.2085	0.200	94.923	2
4	vital signs issues	0.2389	0.220	91.089	3
5	Insulated bucket truck issues (distance, grounding)	0.3529	0.243	67.858	6
6	has strong winds	0.4256	0.342	79.357	4
7	Protective equipment does not meet requirements	1.1885	0.197	15.576	8
8	has no protective measures	2.1385	0.812	36.971	7
Setting up insulation shielding stage—Removal and departure stage					
1	Leakage current from charged components was not detected	0.2149	0.727	337.297	1
2	Leakage caused by severe weather conditions, such as thunderstorms, lightning, snow, fog, and rain	0.3059	0.275	88.899	2
3	Improper operation caused a short circuit	0.7575	0.416	53.917	4
4	vital signs issues	0.2129	0.111	51.137	5
5	Insulated bucket truck issues (distance, grounding)	0.2800	0.112	39	7
6	has strong winds	0.8101	0.370	44.673	6
7	Protective equipment does not meet requirements	0.5600	0.344	60.429	3
8	has no protective measures	7.3753	0.971	12.166	8
Branch line construction stage					
1	Leakage was not detected	0.1618	0.692	426.689	1
2	Leakage caused by severe weather conditions such as thunderstorms, lightning, snow, fog, and rain	0.2806	0.310	109.478	2
3	Improper operation caused a short circuit	0.3464	0.152	42.88	6
4	vital signs issues	0.1961	0.0998	49.892	4
5	insulated bucket truck issues (distance, grounding)	0.3499	0.132	36.725	8
6	has strong winds	0.4558	0.177	37.83	7
7	insulation shielding is substandard	0.9528	0.453	73.789	3
8	Protective equipment does not meet requirements	0.2985	0.0970	31.496	9
9	has no protective measures	1.2114	0.906	46.544	5

### 3.4 Construction of the motivational logic relationship

The establishment of the driving force logic relationship is based on key risk factors, integrating the impact of risk factors with control measures. Through situational analysis of scenario states, treatment methods, and treatment objectives, the development trend of risk factors is dynamically deduced, and tables and diagrams of the driving force logic relationships at each stage are drawn. When severe weather is selected as the driving factor in the driving force logic relationship of “10 kV overhead line energized branch connection line lead wire,” the letters S, M, and T represent scenario state, treatment method, and treatment objective, respectively.

**Fig 7:**
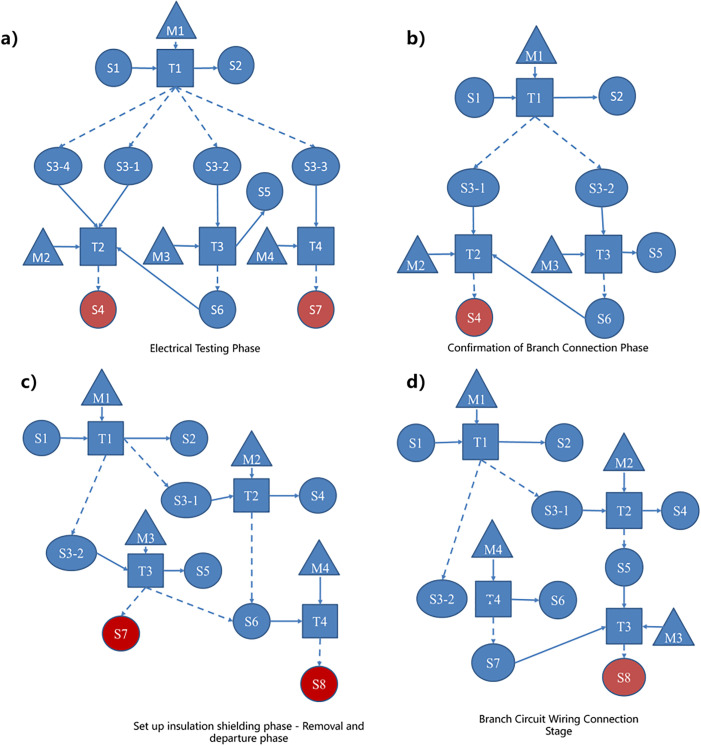
Logic diagram of driving forces during the four stages.

Table 8 illustrates the dynamic logical linkages initiated by extreme weather. The power testing stage exemplifies the risk management situations pertinent to the power testing procedure. This document delineates the response strategies for various risk scenarios at this level, based on the interplay of scenario state (S), treatment method (M), and treatment target (T). Scenario state (S) encompasses many risk scenarios induced by extreme weather during the power testing phase, including work cessation, electric shock, and equipment insulation failure. The treatment method (M) delineates response procedures for various scenarios, encompassing continuous weather surveillance, augmented staff protection, vital sign assessment, and heightened worker alertness. The treatment target (T) delineates the outcomes to be attained for each category of reaction measure, including work suspension/postponement, accident prevention, equipment replacement, and troubleshooting. In the event of severe weather, ongoing weather surveillance is essential. Upon detection of an anomaly, the purpose is to “T1 suspend operations or modify work arrangements in accordance with weather forecast outcomes.” When the “S4 Electric Shock Accident” scenario arises, the appropriate response mechanism and aim are aligned to provide dynamic risk management. The logical relationship is illustrated in a diagram, as depicted in Fig 7.

**Table 8:** Logical relationship of driving factors at each stage starting with severe weather.

Electrical testing stage		
Scenario State S	Treatment method M	Treatment target T
S1. Inclement weather (wind, temperature, humidity)	M1. Continue meteorological monitoring	T1. Suspend operations or postpone operations based on weather forecasts.
S2. Suspend operations		
S3-1. Strong winds may cause people to come into contact with live electrical conductors.	M2. Enhance the personal protection of workers.	T2. Prevent accidents from occurring.
S4. An electric shock accident occurs.		
S3-2. Severe weather caused problems with vital signs.	M3. Continuously monitors vital signs and issues an alarm if they exceed thresholds.	T3. Switch to role B to continue the work, or terminate or postpone the work.
S5. Suspend the task or continue to complete the task.		
S6. Improper or incorrect operation leading to contact with an electrified object.	M2. Enhance the personal protection of workers.	T2. Prevent accidents from occurring.
S4. An electric shock accident occurs.		
S3-3. High humidity causes insulation failure in charged components.	M4. Increase the vigilance of operators and combine multiple voltage testing methods for detection.	T4. Suspend work, investigate the cause of the energization, and then make a decision.
S7. Leakage from a live conductor not detected.		
S3-4 High humidity can cause insulation failure in voltage detectors, leading to contact with live parts.	M2. Enhance the personal protection of workers.	T2. Prevent accidents from occurring.
S4. An electric shock accident occurs.		
**Confirmation of the logical relationship table of the driving forces in the branch line stage**		
Scenario State S	Treatment method M	Treatment target T
S1. Inclement weather (wind, temperature, humidity)	M1. Continue meteorological monitoring	T1. Suspend operations or postpone operations based on weather forecasts.
S2. Suspend operations		
S3-1. Strong winds may cause people to come into contact with live electrical conductors.	M2. Enhance the personal protection of workers.	T2. Prevent accidents from occurring.
S4. An electric shock accident occurs.		
S3-2. Severe weather caused problems with vital signs.	M3. Continuously monitors vital signs and issues an alarm if they exceed thresholds.	T3. Switch to role B to continue the work, or terminate or postpone the work.
S5. Suspend the task or continue to complete the task.		
S6. Contact with a live conductor due to improper or incorrect operation.	M2. Enhance the personal protection of workers.	T2. Prevent accidents from occurring.
S3. An electric shock accident occurs.		
**Setting up the insulation shielding stage**		
Scenario State S	Treatment method M	Treatment target T
S1. Inclement weather (wind, temperature, humidity)	M1. Continue meteorological monitoring	T1. Suspend operations or postpone operations based on weather forecasts.
S2. Suspend operations		
S3-1. High humidity caused leakage of live parts, which was not detected during the voltage testing phase.	M2. Increase the vigilance of operators and combine multiple voltage testing methods for detection.	T2. Suspend work, investigate the cause of the energization, and then make a decision.
S4. Complete the work after repairing the leakage.		
S3-2. Severe weather caused problems with vital signs.	M3. Continuously monitors vital signs and issues an alarm if they exceed thresholds.	T3. Switch to role B to continue the work, or terminate or postpone the work.
S5. Suspend the task or continue to complete the task.		
S6. Contact with a live conductor due to improper or incorrect operation.	M4. Enhance worker personal protection	T4. Prevent accidents from occurring.
S7. Improper or incorrect operation leading to failure in the insulation shielding stage.		
S8. An electric accident occurs.		
**Branch line splicing stage**		
Scenario State S	Treatment method M	Treatment target T
S1. Inclement weather (wind, temperature, humidity)	M1. Continue meteorological monitoring	T1. Suspend operations or postpone operations based on weather forecasts.
S2. Suspend operations		
S3-1. High humidity caused leakage of live parts, which was not detected during the voltage testing phase.	M2. Increase the vigilance of operators and combine multiple voltage testing methods for detection.	T2. Suspend work, investigate the cause of the energization, and then make a decision.
S4. Complete the work after repairing the leakage.		
S5. Inadequate insulation shielding caused workers to come into contact with live parts.	M3. Enhance worker personal protection	T3. Prevent accidents from occurring.
S3-2. Severe weather caused problems with vital signs.	M4. Continuously monitors vital signs and issues an alarm if they exceed thresholds.	T4. Switch to role B to continue the work, or terminate or postpone the work.
S6. Suspend the work or continue to complete the work.		
S7. Contact with a live conductor due to improper or incorrect operation.	M3. Enhance worker personal protection	T3. Prevent accidents from occurring.
S8. An electric shock accident occurs.		

## 4. Discussion

This paper combines the grey relational analysis method and Bayesian network model to conduct a comprehensive risk assessment of the live-line operation for connecting branch leads on 10 kV overhead lines. Among them, grey relational analysis is used to objectively analyze the correlation of risk factors and determine key indicators; the Bayesian network quantifies the weight of each risk factor based on expert experience. The combination of the two methods can effectively improve the accuracy and reliability of the assessment results.

In the voltage testing stage, factors such as electricity leakage caused by long-term service of live components, insulation failure of voltage testers due to thunderstorms, rain, snow, fog and other harsh environments, and lack of protective measures have relatively high comprehensive weights. Therefore, before operation, priority should be given to checking severe weather and high-humidity environments, and personal protection must be strictly implemented to prevent electric shock accidents.

In the branch line confirmation stage, in addition to missed voltage testing, illegal operation at the rear end and physical and mental health problems of operators become the main risks, leading to an obvious change in the risk ranking. Since the incidence of operation without protection is low in this stage, the weight of related risks decreases; however, accidents caused by violations due to physical and mental health problems of operators occur frequently. Therefore, during operation, environmental factors should be considered, supervision and physical and mental health checks of operators should be strengthened, and illegal operations should be eliminated.

In the stages of insulation shielding installation, removal and departure, in addition to missed voltage testing, the impacts of harsh environments and unqualified protective equipment increase significantly, while the risk weight of lacking protective measures decreases somewhat, indicating that operators have relatively strong awareness of prevention at this stage. Nevertheless, environmental monitoring should be enhanced and the compliance of protective equipment must be strictly verified.

In the branch line connection stage, unqualified insulation shielding rises to a major risk factor. This shows that, besides severe weather and high-humidity environments, the operation quality of the previous procedure is critical to the safety of this stage. Strict control over the preceding operation process is required to prevent electric shock accidents.

The calculation results show that human factors and environmental factors are the main risk sources, which are basically consistent with the patterns of historical accidents. Two important conclusions are also drawn: (1) the physical and mental health of operators has a prominent weight and should be highly valued, Although physical and mental health is widely recognized as an important factor affecting workers’ safety performance, it is often under-emphasized in current power industry safety management. This is mainly because traditional safety management focuses on equipment reliability, operational compliance, and accident prevention, while factors such as psychological stress, fatigue accumulation, sleep quality, and mental workload are difficult to quantify and monitor. Moreover, existing safety performance indicators are primarily accident- and equipment-oriented, resulting in insufficient consideration of workers’ physical and mental conditions in safety assessment and decision-making. To address this issue, power enterprises should establish regular physical and psychological health assessment mechanisms, utilize wearable devices and digital technologies to monitor fatigue and physiological indicators in real time, optimize work schedules to reduce excessive overtime and ensure adequate recovery periods, provide psychological counseling and resilience training, and integrate physical and mental health indicators into safety performance evaluation systems. These measures can facilitate the early identification and mitigation of human-related risks, thereby improving the overall effectiveness of safety management. (2) From the weight calculation and logical relationship analysis, a significant transmission effect can be observed among different operational procedures, indicating that the quality and safety performance of preceding steps have a direct and cumulative influence on subsequent operations. Deficiencies, omissions, or latent risks generated in earlier procedures may propagate through the workflow and amplify safety risks in later stages, thereby increasing the likelihood of operational errors and accidents. Therefore, safety management should not focus solely on individual procedures but should emphasize the continuity and interdependence of the entire operational process. It is recommended that a comprehensive re-assessment be conducted after the completion of each procedure to verify compliance with safety requirements, identify newly emerging risks, and ensure that all necessary control measures remain effective before proceeding to the next stage. Such a dynamic verification mechanism can help interrupt risk transmission pathways and enhance the overall safety and reliability of power operations.

## 5. Conclusion

1) A risk assessment method for power workers based on the BN analyzes risks from two perspectives: the probability of accidents and the magnitude of the impact of risk factors. It constructs a causal logic relationship to clarify the dynamic evolution of accidents. This method is applicable to risk assessment and evolution in power operations, helping to fully protect the personal safety of power workers and deeply trace the root causes and responsibilities of accidents.
2) The construction of typical live-line work scenarios and risk assessment results show that the magnitude of the impact of risk factors varies at different stages of the same power operation. To provide sufficient safety guarantees for all power workers, it is necessary to conduct in-depth and detailed scenario construction and risk analysis for each power operation, accurately identify risk factors at key stages, and strengthen the control of risk factors with significant impact.
3) The causal logic relationship demonstrates the corresponding measures that should be taken in a timely manner during the accident process, as well as the impact of human intervention. Effective control of risk factors at any stage will significantly reduce the probability of accidents.
4) The results of this research can be applied to the precise management of the safety of power workers and even extended to the safety management of other industries such as chemical operations. On the other hand, it can serve as a core foundation to lay a key data foundation for the deep integration with artificial intelligence technology in the future, help to accurately identify and scientifically assess the role mechanism and impact of risk factors in key links of operations in various industries, and ultimately achieve real-time early warning and dynamic response to potential risks, providing data-driven intelligent solutions for safety management.

## Supporting information

S1 File Support information. (ZIP)
